# Machine Learning-based Development and Validation of a Cell Senescence Predictive and Prognostic Signature in Intrahepatic Cholangiocarcinoma

**DOI:** 10.7150/jca.92698

**Published:** 2024-03-25

**Authors:** Ruida Yang, Feidi Sun, Yu Shi, Huanhuan Wang, Yangwei Fan, Yinying Wu, Ruihan Fan, Shaobo Wu, Liankang Sun

**Affiliations:** 1 Department of Hepatobiliary Surgery, The First Affiliated Hospital of Xi'an Jiaotong University, Xi'an 710061, PR China.; 2 Department of Oncology, the First Affiliated Hospital of Xi'an Jiaotong University, Xi'an 710061, PR China.

**Keywords:** cellular senescence, cholangiocarcinoma, machine learning, long non-coding RNAs (lncRNAs), signature

## Abstract

**Background:** Previous studies have shown that cellular senescence is strongly associated with tumorigenesis and the tumor microenvironment. Accordingly, we developed a novel prognostic signature for intrahepatic cholangiocarcinoma (ICCA) based on senescence-associated long non-coding RNAs (SR-lncRNAs) and identified a lncRNA-miRNA-mRNA axis involving in ICCA.

**Methods:** Based on the 197 senescence-associated genes (SRGs) from Genacards and their expression in Fu-ICCA cohort, we identified 20 lncRNAs as senescence-associated lncRNAs (SR-lncRNAs) through co-expression and cox-regression analysis. According to 20 SR-lncRNAs, patients with ICCA were classified into 2 molecular subtypes using unsupervised clustering machine learning approach and to explore the prognostic and functional heterogeneity between these two subtypes. Subsequently, we integrated 113 machine learning algorithms to develop senescence-related lncRNA signature, ultimately identifying 11 lncRNAs and constructing prognostic models and risk stratification. The correlation between the signature and the immune landscape, immunotherapy response as well as drug sensitivity are explored too.

**Results**: We developed a novel senescence related signature. The predictive model and risk score calculated by the signature exhibited favorable prognostic predictive performance, which is a suitable independent risk factor for the prognosis of patients with ICCA based on Kaplan-Meier plotter, nomogram and receiving operating characteristic (ROC) curves. The results were validated using external datasets. Estimate, ssGSEA (single sample gene set enrichment analysis), IPS (immunophenotype score) and TIDE (tumor immune dysfunction and exclusion) algorithms revealed higher immune infiltration, higher immune scores, lower immune escape potential and better response to immunotherapy in the high-risk group. In addition, signature identifies eight chemotherapeutic agents, including cisplatin for patients with different risk levels, providing guidance for clinical treatment. Finally, we identified a set of lncRNA-miRNA-mRNA axes involved in ICCA through regulation of senescence.

**Conclusion:** SR-lncRNAs signature can favorably predict the prognosis, risk stratification, immune landscape and immunotherapy response of patients with ICCA and consequently guide individualized treatment.

## Introduction

Cholangiocarcinoma (CCA) is a rare cancer that develops from epithelial cells in the biliary duct 1. It can be categorized into three types depending on where it is located in the body: intrahepatic, perihilar, and distal cholangiocarcinoma 2. Among the primary malignant liver cancers, the second most common one is intrahepatic cholangiocarcinoma (ICCA), which arises from the biliary epithelium present in the liver 3. It is more likely to be diagnosed in patients with normal liver function or underlying liver disease 1. Surgical treatment is the preferred option for patients with ICCA, while chemotherapy, usually consisting of gemcitabine and cisplatin, is often used for inoperable cases. Despite the significant advancements in treatment approaches, patients with unresectable ICCA typically have a gloomy prognosis, with a survival rate of under 12 months 4. This emphasizes the necessity of conducting additional studies on the mechanism of ICCA to enhance the available treatment choices and increase the chances of survival for patients.

Cellular senescence is a phenomenon characterized by cell cycle arrest and a limited lifespan of proliferation. It includes replicative senescence caused by telomere shortening, as well as premature senescence triggered by oncogene activation and DNA damage response. These processes play crucial roles in various stages of tumorigenesis 5. Scientific evidence clearly demonstrates that the activation of oncogenes in mammalian cells causes proliferative stress and gives rise to senescence, effectively blocking the transition of benign tumor lesions into malignancies 6. On the one hand, studies have demonstrated that mutations in oncogenes like RAS, as well as overexpression of HER2, EGFR, and PI3K, contribute to driving senescence in tumor cells 5. On the other hand, what's special is that senescent cells could secrete numerous proinflammatory cytokines, chemokines, growth factors, and proteases, which can exert a wide range of effects on adjacent cells. This specific phenotype exhibited by senescent cells is referred to as the senescence-associated secretory phenotype (SASP). The effect of SASP on tumor remains controversial 7. Moreover, there is a growing body of evidence indicating that the advancement of human cancer may be influenced by long non-coding RNAs (lncRNAs) through the encoding of peptides with biological functions. Specifically, a search has indicated that PINT87aa, a certain lncRNA, might induce cellular senescence in hepatocellular carcinoma 8. Besides, a former study had indicated that abnormal molecular mechanisms associated with cellular senescence might be triggering factors to tumorigenesis of ICCA, which included DNA damage repair, proactivation of pro-inflammatory cytokines and abnormal tumor microenvironment 9. Nevertheless, the functions of the majority of lncRNA between cellular senescence and ICCA remain unclear.

Hence, this study aims to investigate the role of long non-coding RNAs (lncRNAs) between cellular senescence and ICCA. 20 lncRNAs as SR-lncRNAs were identified based on several cohorts. ICCA patients were classified by unsupervised clustering based on SR-lncRNAs and the heterogeneity between two sub-types were explored. Integrating 113 machine learning algorithms, we developed a senescence-related lncRNA signature, ultimately identifying 11 lncRNAs and constructing prognostic models. The model favourably enhanced our ability to accurately forecast the prognosis and immunotherapy response of patients with ICCA. Moreover, the findings of this study revealed a substantial correlation between the established signature and the tumor immune microenvironment. Further, we found that a lncRNA-miRNA-mRNA axis is involved in ICCA through influencing cellular senescence. By doing so, it confirmed the significant involvement of cellular senescence in ICCA, offering theoretical backing for more accurate and readily available treatment alternatives for patients with ICCA.

## Materials and Methods

### Acquisition and preprocessing of data

The NODE (National Omics Data Encyclopedia) 10 provided the TPM transcriptome data and complete clinical annotations for 255 patients with ICCA. The senescence-related genes was obtained from the Genecards database 11. 244 cases were used as the internal cohort after excluding patients who did not have survival status. Moreover, we selected validation cohort consisting of 44 patients with CHOL from the Cancer Genome Atlas (TCGA), 52 patients from the GSE201241 and 23 patients from GSE32958 12.

### Identification of key lncRNAs

We first conducted co-expression analysis with a filter of p value = 0.001 and correlation = 0.4 to identify lncRNAs that were co-expressed with 197 senescence-related genes in Fu-ICCA cohort. Afterwards, we employed univariate Cox regression analysis on selected lncRNAs associated with senescence, with the aim of assessing their prognostic importance. Ultimately, 19 lncRNAs were identified as SR-lncRNAs. Next, we demonstrated SR-lncRNAS and SRGs network maps using cytoscape (Fig 2C) (https://cn.string-db.org) 13.

### Consensus clustering analysis and construction of gene clusters

We conducted unsupervised K-means clustering analysis to determine if senescence-related genes could classify ICCA patients. In order to classify the ICCA patients into subtypes, we employed the "ConsensusClusterPlus" package in the R software 14. The package was used for conducting consensus clustering analysis on the expression values of crucial lncRNAs in every sample of the FU-iCCA cohort, ensuring verification through 1,000 iterations. The validation of the determination of clusters was carried out after analyzing the CDF curve 15. The Kaplan-Meier survival curve and the Log-Rank algorithm were applied for assessing the survival probability of the two clusters. We also utilized the R package "pheatmap" to construct a heatmap displaying the connection between the expression of the 19 lncRNAs and the clinical features in the two clusters 16.

### Immune characteristics and biological function in both clusters

To further elucidate the heterogeneity of the two clusters in terms of their immune landscape and biological functions, several algorithms were integrated. The "xCell" R package used to compute the stromal score, immune score, and microenvironment score for every ICCA sample 17. Furthermore, the proportions of the immune cells in total of 22 in each cluster were calculated via the package "CIBERSORT" 18. The immune infiltration abundance of 9 immune cells in two clusters was also evaluated using the “EPIC” algorithm 19. The estimated immune cell type scores in each sample summed up to 1. The application of the R package "GSVA" was allowed for the enrichment of Gene Set Variation Analysis (GSVA) in order to explore the variability of various biological processes 20. Subsequently, functional annotation was carried out using the R package "ClusterProfiler" 21.

### Integrative construction of a senescence signature and prognostic risk model

Based on the expression profiles of SR-lncRNAs. These lncRNAs were subjected to our machine learning-based integrative procedure to develop a consensus senescence-related lncRNA signature (SRLS). Utilizing Fu-ICCA, we fitted 113 kinds of prediction models via the LOOCV (Leave One Out Cross-Validation) framework and further calculated the AUC of each model across all validation datasets. We excluded models with less than five outputs for the number of feature genes. The most optimal model was selected and further prognostic modelling and risk stratification was established based on the signature genes output from this model. Thus, the least absolute shrinkage and selection operator (LASSO) algorithm (when partial likelihood deviance is at its lowest) and generalized linear regression analysis were used to further filter SR-lncRNAs in the training cohort. Finally, the SRLS including 11 lncRNAs was obtained.

### Risk stratification and model validation

We developed a new risk score for patients with ICCA based on the signature. The risk score was calculated as the sum of the weighted expression of individual genes as follows: risk score = Σ (expression (Gene_n_) × coefficient (Gene_n_)) (where expression (Gene_n_) represents the expression of a specific gene and coefficient (Gene_n_) is its corresponding coefficient). We assigned the 244 ICCA patients into two groups based on the risk scores. Based on the median risk score, the 244 ICCA patients in the FU-iCCA cohort were divided into distinct clinical groups, which were named low-risk and high-risk groups. In addition, we divided the 244 patients into two groups, the internal train cohort and the test cohort, in a random manner. The OS of ICCA patients in both groups was compared by the Kaplan-Meier survival curve. Following that, the performance of the risk model in predicting outcomes was assessed through the "survival ROC" function in R 22. Subsequently, we performed univariate and multivariate Cox regression analyses to examine the association between intrahepatic metastasis, HBsAg (hepatitis B surface antigen) level, vascular invasion, risk score, regional lymph node metastasis, distal metastasis, perineural invasion, TNM stage, and overall survival (OS) in patients with ICCA. Our aim was to identify independent predictors of OS. Moreover, we conducted a mutation landscape with the purpose of examining the mutation patterns in both the high- and low-risk score groups.

### Prediction of immune landscape, immunotherapy response, stemness, and chemosensitivity

To examine how immune infiltration affects tumor development and treatment, we have integrated multiple immune infiltration algorithms. We evaluated the immunophenotype score (IPS), Estimate score, immune checkpoints in both high and low risk samples 23.

The TIDE algorithm was employed to predict the response to immunotherapy by utilizing Tumor Immune Dysfunction and Exclusion (TIDE), Exclusion, and Dysfunction, and Microsatellite Instability (MSI) scores.

The mRNA Stemness index (mRNAsi) is an index calculated based on expression data describing the similarity of tumor cells to stem cells with closer to 1 indicating lower differentiation and stronger stem cell characteristics 24. The cell expression data were trained with the OCLR (one-class logistic regression) machine learning algorithm, and a cell stemness model was constructed using the “gelnet” R package 25, 26. Based on the obtained stemness model, mRNAsi was calculated for Fu-ICCA data.

The R package "pRRophetic" was utilized to predict the half-maximal inhibitory concentration (IC50) of drugs.

### Identification of hub lncRNA-miRNA-mRNA axis involving in senescence in ICCA

Differentially expressed genes between risk subgroups were found based on “limma” package 27. Differentially expressed genes were categorized as genes with a P-value lower than 0.05. We performed GSEA analyses to annotate different biological functions based on ranked differentially expressed genes for risk subgroups separately.

To personalize and specifically validate the involvement of SRLS in ICCA, we identified target miRNAs for ADAMTSL4-AS1. Particularly, a previous study has shown that miR-214-3p is involved in cellular senescence 28. Then, we predicted the target mRNAs of the miRNAs and specifically selected senescence-related targets. Tumor samples from the TCGA-CHOL cohort were used to assess lncRNA-miRNA, miRNA-mRNA correlations and regression equation calculations. MirNet and ENCORI were used for lncRNA-miRNA and miRNA-mRNA prediction respectively 29, 30.

### Statistical analysis

The study's statistical analysis was conducted using R-4.1.2 software and its support packages. The statistical significance of variables that were distributed was estimated through the Student's t-test, while for variables that were not normally distributed, the Wilcoxon rank sum test was used. We carried the Kruskal-Wallis H-test, Dunn's test, and Wilcoxon rank sum test to assess the disparities in clinicopathological features between the low-risk and high-risk groups. Furthermore, the Chi-square test was utilized to analyze variations in clinicopathological characteristics between the two molecular subtypes. The Kaplan-Meier survival curve and Log-Rank test were applied to analyze the survival of ICCA patients. To ascertain independent predictors of overall survival in ICCA patients, multivariate Cox regression analysis was conducted. Correlation coefficients were determined by Spearman correlation, and a P-value < 0.05 was deemed statistically significant.

## Results

### Identification of SR-lncRNAs

**Figure 1:**
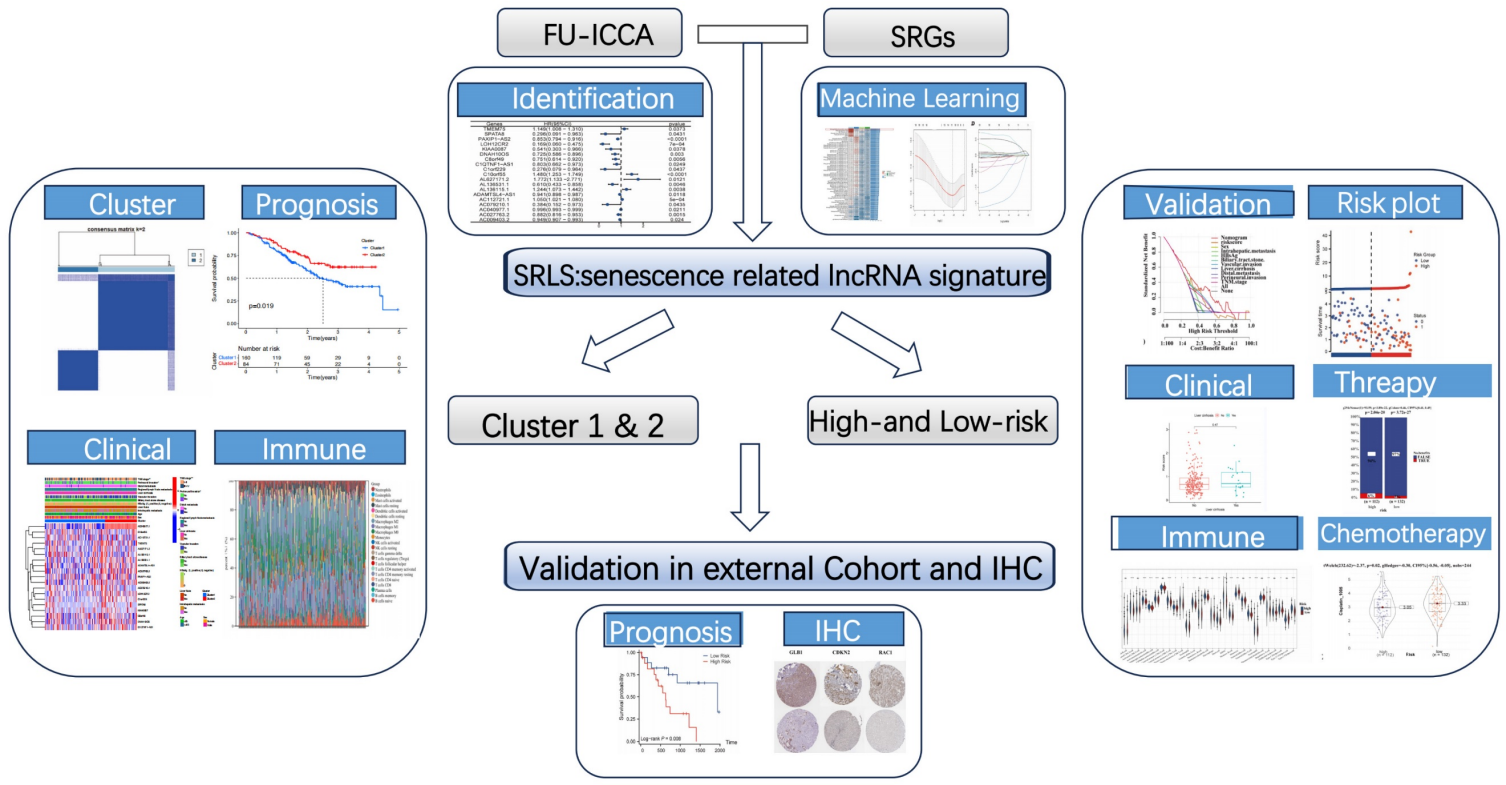
The graphical abstract of study.

**Figure 2:**
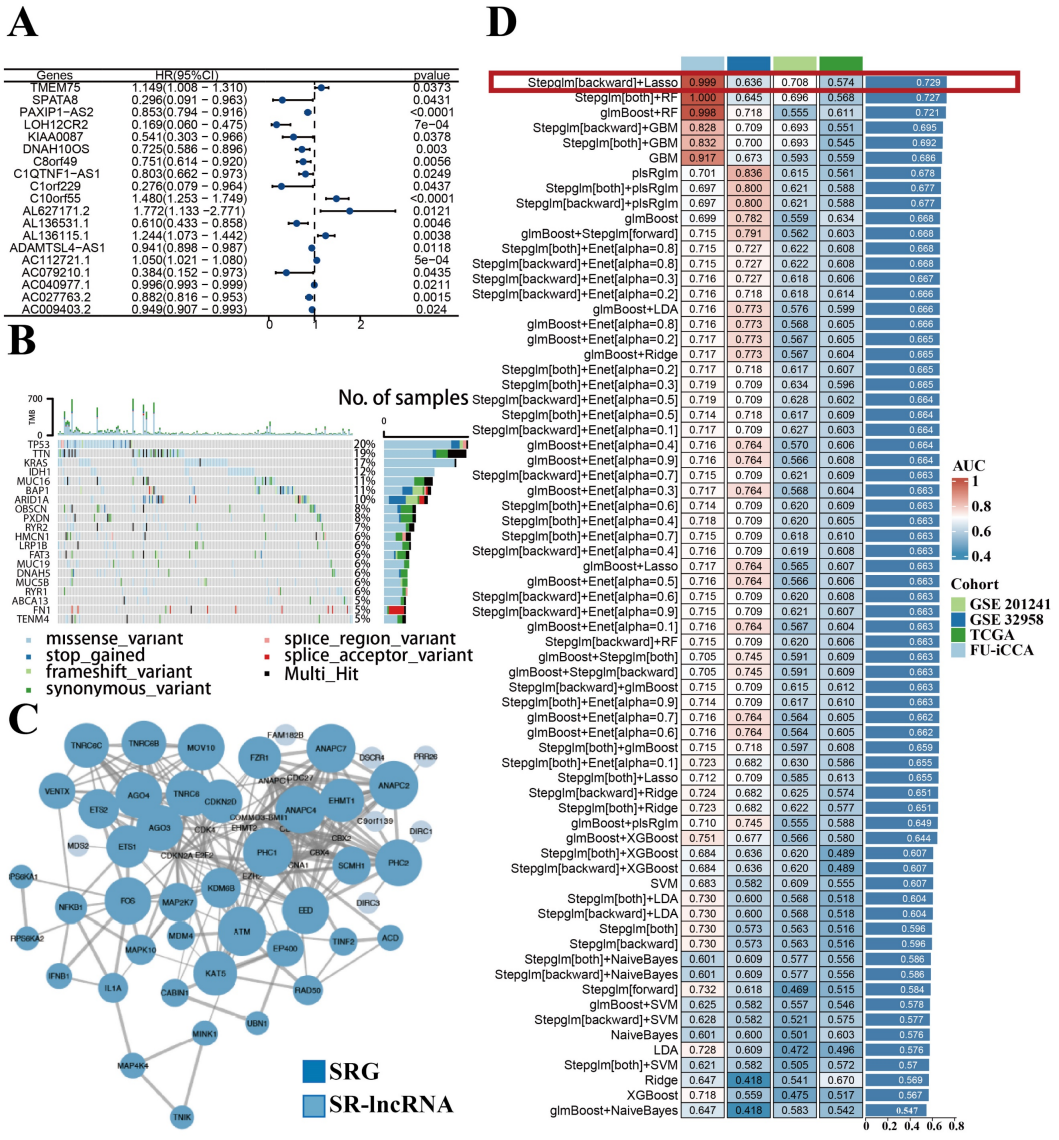
Identification of SR-lncRNAs and integration of 113 machine learning algorithms. (A) Cox regression identified 20 SR-lncRNAs. (B) Mutational landscape of the Fu-ICCA cohort. (C) Interaction network diagram of SRG and SR-lncRNAs. (D) Integration of 113 machine learning algorithms to output optimal SRLS. SR-lncRNAs: senescence-related lncRNAs; SRG: senescence-related gene; SRLS: senescence-related lncRNA signature.

The flow chart presented the overall design of our study (Figure 1). We obtained 197 senescence-related genes from Genecards. Figure 2A displays the contribution of 19 SR-lncRNAs to OS that were identified based on co-expressed and univariate Cox regression analysis in Fu-ICCA cohort. The mutation landscape of Fu-ICCA was represented in oncoplot, which suggests that missense mutations are the most common pattern, with TP53 having the highest mutation rate of 20% (Figure 2B). The network of co-expressed mRNAs and lncRNAs is depicted in Figure 2C. This network reveals interactions relationships between SR-lncRNAs and the 197 senescence-related genes.

### Construction of distinct molecular clusters

**Figure 3:**
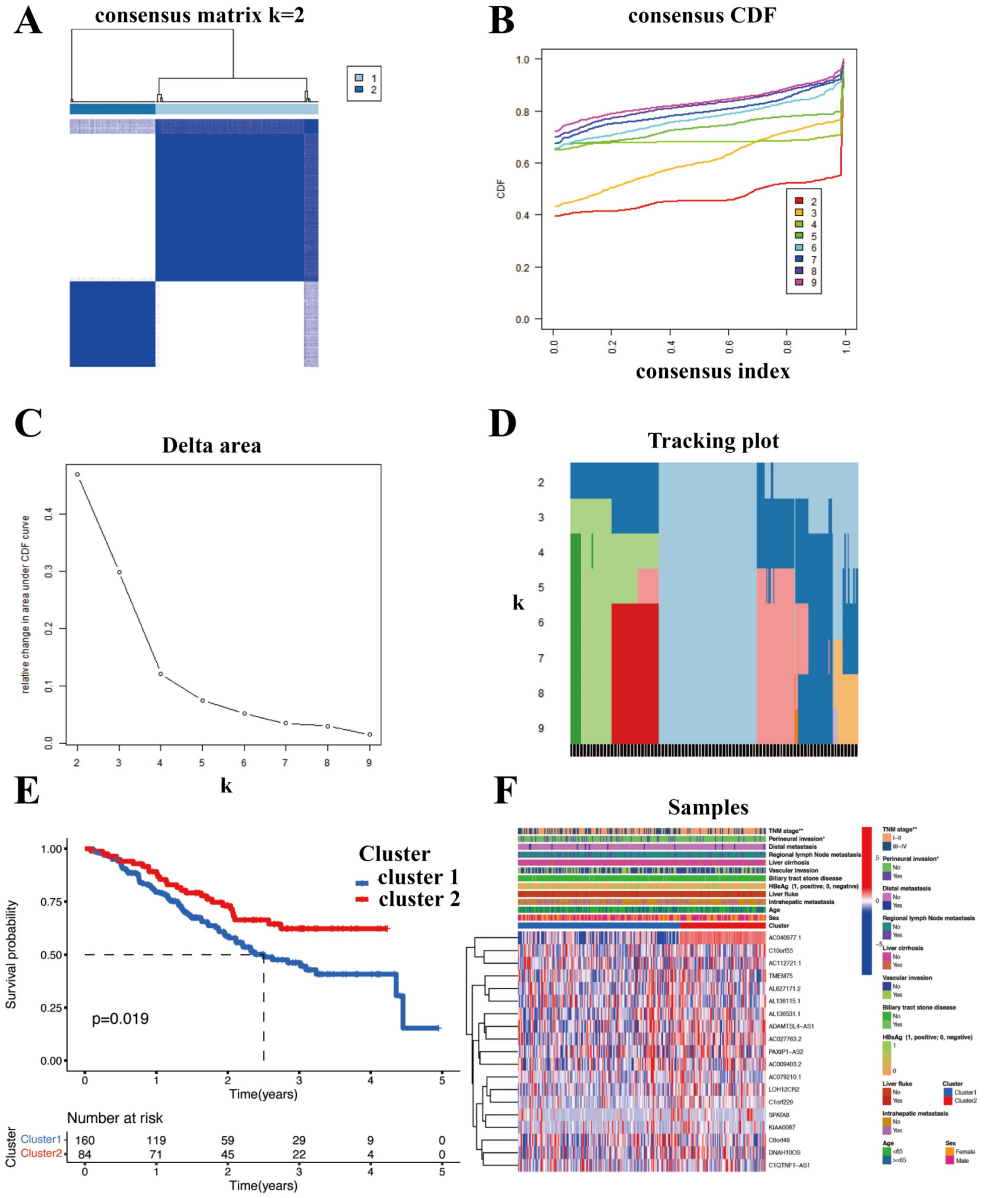
Construction of distinct molecular clusters. (A) Based on unsupervised clustering 244 patients with ICCA were stratified into 2 distinct subgroups. (B, C and D) The consensus CDF and the relative change in the area below the CDF curve confirmed that the consensus matrix represents a value of k = 2. (E) OS of different clusters. (F) Clinical characterization and heterogeneity of SR-lncRNAs expression between different clusters. OS, overall survival.

With the 1000 iterations of hierarchical clustering of 244 ICCA samples from NODE for k =2~5, we found that those samples showed the highest consistency when k=2. On the basis of such result, we preliminarily stratified the 244 ICCA patients into 2 distinct subgroups (Figure 3A). Moreover, based on the outcomes of the consensus CDF and the relative change in the area below the CDF curve, we confirmed that the consensus matrix represents a value of k=2, indicating the number of subgroups (Figure 3B; Figure 3C; Figure 3D). In cluster 2, survival analysis indicated a favorable prognosis for patients, while cluster 1 experienced a poor outcome (Figure 3E). Patients in the two clusters also showed differences in clinical features, such as more advanced TNM staging and more frequent occurrence of perineural invasion in cluster 1 patients (Figure 3F).

### Characteristic of immune and biological function within two clusters

**Figure 4:**
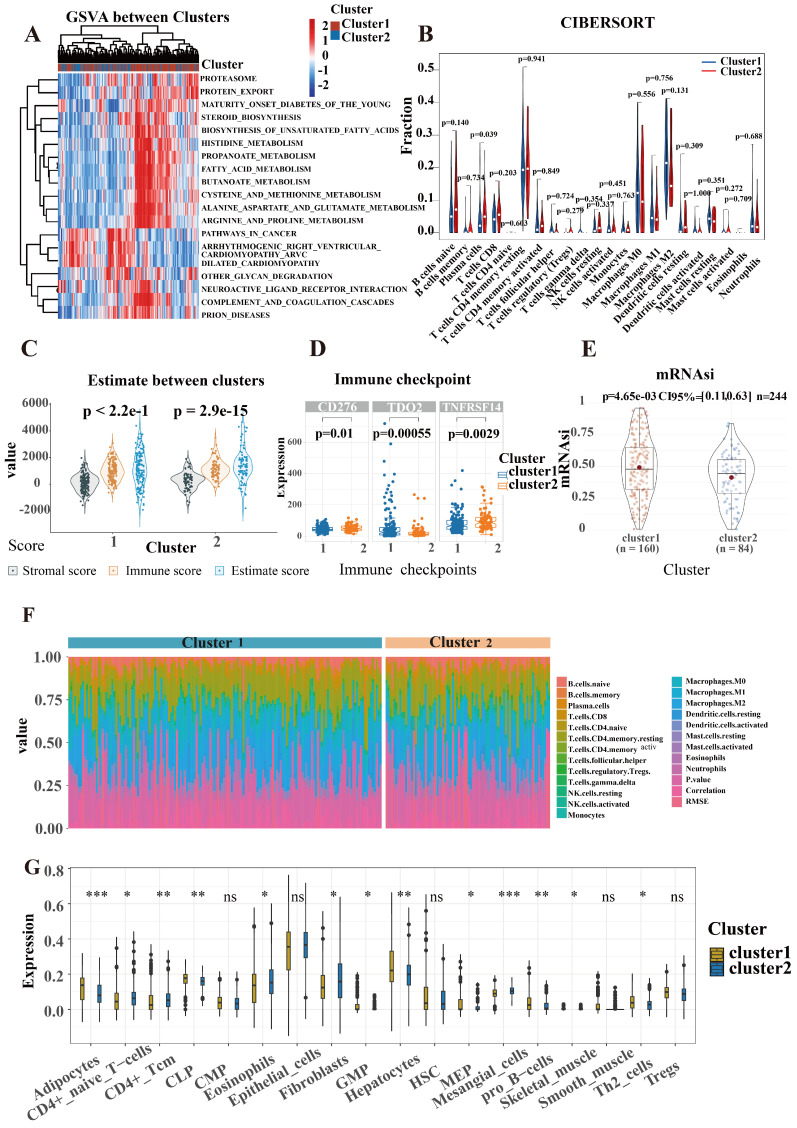
Characteristic of immune and biological function within two clusters. (A) GSVA illustrated the up-regulated and down-regulated pathways in cluster1 and cluster2. (B) CIBERSORT analysis to examine the relative ratios of 22 immune cells. (C) Estimate algorithm of ICCA patients was conducted to calculate estimate score. (D) A significant difference in three immune checkpoint markers between two clusters. (E) The associations between the mRNAsi and the clusters. (F) The proportion of each cell is shown in CIBERSORT. (G) The differences of immune cells between clusters using the Xcell algorithm.

SR-lncRNAs are almost always differentially expressed between clusters (Figure S1). Next, the biological functions of SR-lncRNA molecular subtypes were examined through GSVA. Figure 4A illustrates the up-regulated and down-regulated pathways in cluster1 and cluster2. Proteasome pathway, lipid metabolism, and amino acid metabolism significantly upregulated in cluster1 than in cluster2. Increased cell cycle-dependent proteasomes and exacerbated disruption of lipid and amino acid metabolism in aging have been confirmed by previous studies, which suggests that cluster 1 is more characterized than cluster 2 in terms of senescence phenotypes.

To delve deeper into the immune cell composition, we employed the "CIBERSORT" analysis technique to examine the relative ratios of 22 immune cells. Figure 4B demonstrates that plasm cells exhibited the most significant difference in infiltration between the two clusters, with a p-value of 0.039. The proportion of each cell is shown in Figure 4F. In order to figure out the correlation between the clusters and immune micro-environment, estimation algorithm of ICCA patients was conducted, but there is no significance (Figure 4C).

Higher stemness of tumor indicating lower differentiation and stronger stem cell characteristics. Figure 4E shows that cluster1 has stronger stem cell properties and worse tumor differentiation. We also explored the associations between the mRNAsi and the clusters (Figure 4E). Cancers frequently exploit these immune checkpoints as resistance mechanism to evade immune surveillance. We discovered a significant difference in three immune checkpoint markers, with CD276 and TNFRSF 14 showing higher expression in cluster 2 while TDO2 had higher expression in cluster 1(Figure 4D). To validate the robustness and stability of the immune infiltration analysis, we further analyzed the differences of immune cells between clusters using the Xcell algorithm (Figure 4G).

### Construction of SRLS and prognostic model in ICCA

**Figure 5:**
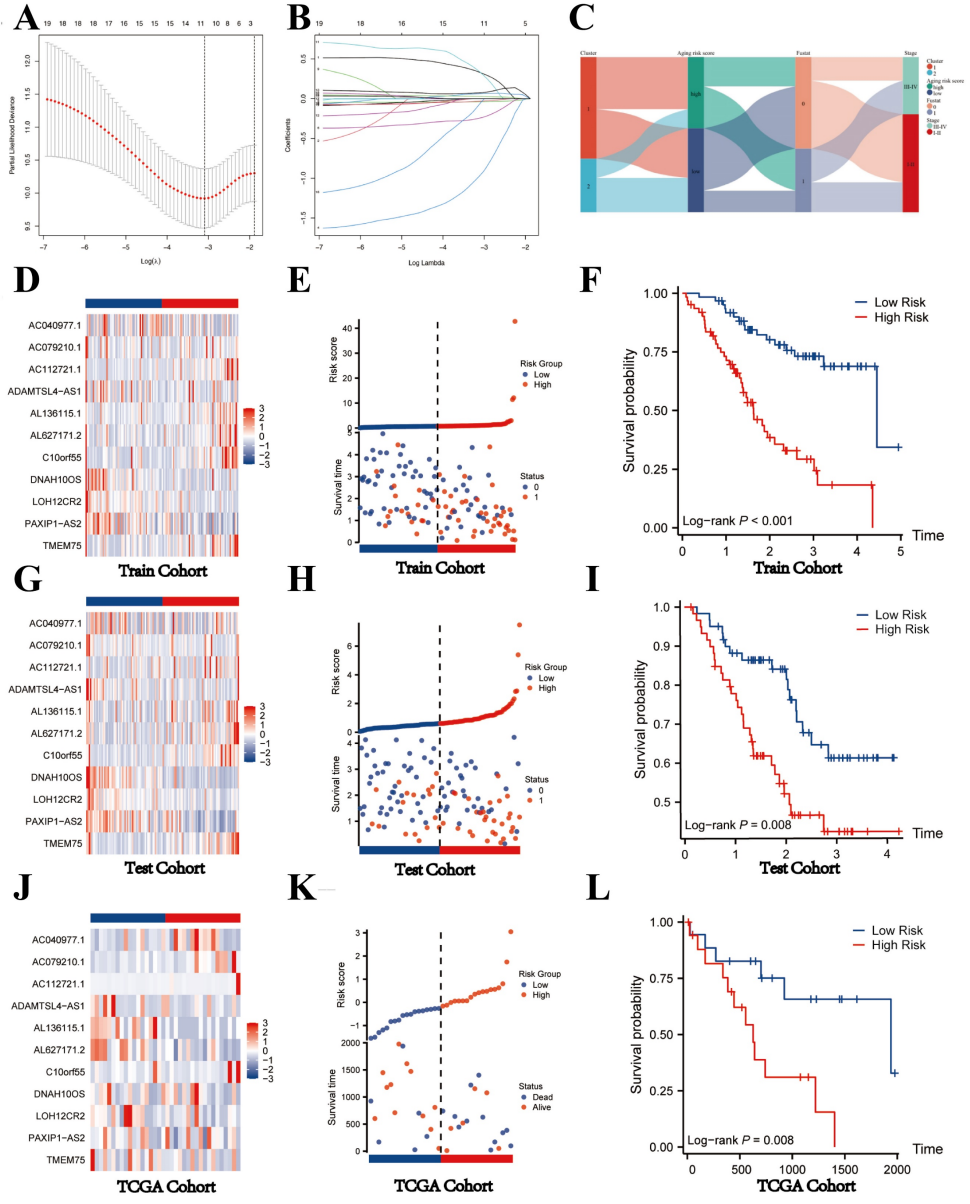
Construction of prognostic model and validation of SRLS in internal and external validation cohort. (A, B) The most optimal model exported 11 lncRNAs identified as SRLS. (C) The correspondence between the different phenotypes. (D, G, J) The expression characteristics of the 11 crucial lncRNAs in our risk model among the low- and high-risk groups in all cohorts. (E, H, K) The risk plots in all cohorts. (F, I, L) The high-risk group had considerably poorer overall survival (OS) when compared to the low-risk group in all cohorts.

The Fu-ICCA dataset was used to fit 113 prediction models using the LOOCV framework. The AUC was calculated for each model across all validation datasets. We show how well the top 70 models with the highest average AUC values predicted the four cohorts (Figure 2D). The most optimal model, stepglm[backward]+lasso, was selected. Thus, the training cohort underwent the least absolute shrinkage and selection operator (LASSO) algorithm, with the lowest partial likelihood deviance, and generalized linear regression analysis to filter SR-lncRNAs further. Then the prognostic model and risk stratification were conducted based on the output signature genes from the most optimal model, resulting in 11 candidate genes with non-zero regression coefficients, which were identified as SRLS (Figure 5A, 5B).

### Validation of SRLS and prognostic model in internal and external validation cohort

After identification, we randomly allocated the 244 patients into 2 groups, 124 cases in training cohort and 120 cases in test cohort. The two groups were strictly classified into low and high-risk categories based on the median risk score. Figure 5C shows the correspondence between the different phenotypes. We found that patients in cluster1 flowed more to the high-risk group, which suggests that the survival characteristics and biological functions of the high-risk group are more consistent with cluster1, which is also in line with the previous analyses.

The heatmaps indicated the expression characteristics of the 11 crucial lncRNAs in our risk model among the low- and high-risk groups in both cohorts (Figure 5D, G). It was observed that the expression of TMEM75, Cr10orf55, AL627171.2, AL136115.1, and AC112721.1 elevate, whereas PAXIP1-AS2, LOH12CR2, DNAH10OS, ADAMTSL4-AS1, AC079210.1, and AC040977.1 displayed low levels of expression in the training cohort and test cohort.

As shown in the risk plots, we found that with the survival time in ICCA patients of training and testing cohort increasing, the number of red dots gradually increased, suggesting that more patients in the high-risk group died (Figure 5E, H). In comparison to the high-risk group, the low-risk group of patients frequently exhibited a longer duration of survival. Patients in the high-risk group had considerably poorer overall survival (OS) when compared to the low-risk group, showing a p-value of less than 0.001 in the training cohort and a p-value of 0.008 in the testing cohort (Figure 5F; Figure 5I).

To further validate the generalizability of the model, we obtained TCGA-CHOL dateset of 44 patients with ICCA as an external validation cohort. After considering the risk score, we categorized the 44 patients into two groups: the high-risk group and the low-risk group. The heatmap shown the expression of 11 key lncRNAs in the two groups. We found that the expression level of TMEM75, PAXIP1-AS2, LOH12CR2, AL627171.2, AL136115.1, and ADAMTSL4-AS1 was relatively abundant in low-risk group, while AC040977.1, AC079210.1, AC112721.1, and C100rf55 was high-expressed in high-risk group, indicating that the expression of the 11 lncRNAs in the TCGA cohort was consistent with that in the internal validation (Figure 5J). Consistent with the internal validation cohort findings, the high-risk group displayed a lower survival rate and a higher likelihood of death, as depicted by the risk plot and Kaplan-Meier plot (Figure 5K,5L). These findings imply that lncRNAs with significant differences in expression levels between high-risk and low-risk groups might have a unique regulatory effect on the contrasting prognoses seen in these two groups.

### Characteristic of Clinical utility and predictive applicability for SRLS

To evaluate the potential clinical utility of SRLS, we also targeted the clinical characteristics of ICCA. Fu-ICCA cohort was subjected to an applicability analysis.

**Figure 6:**
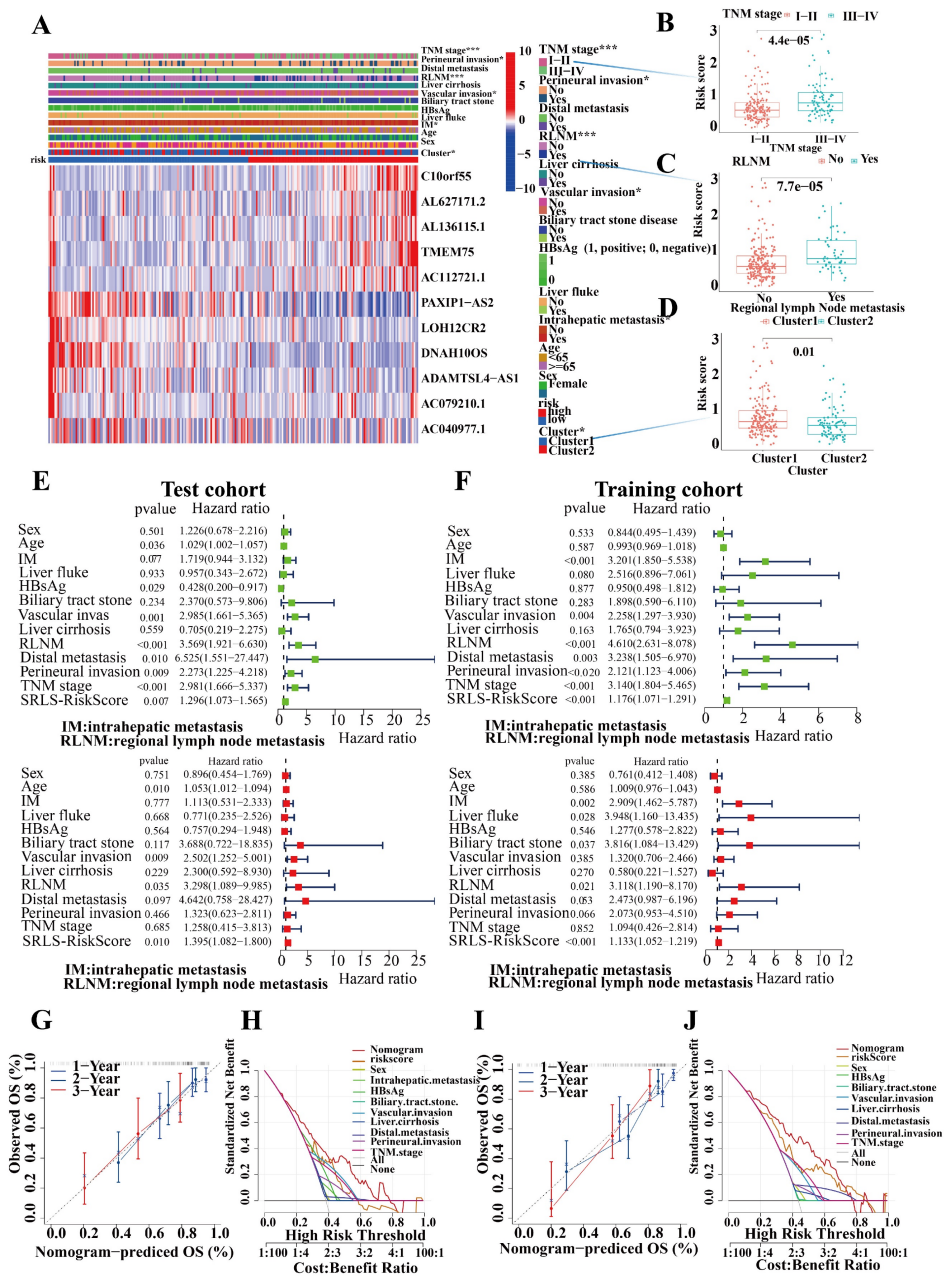
Characteristic of Clinical utility and predictive applicability for SRLS. (A, B, C and D) The occurrence of various clinicopathological factors in both low and high-risk groups. (E, F) Univariate and multivariate Cox regression analyses of risk scores in test and train cohorts were performed to exclude the interference of other clinical factors. (G, I) The calibration curve in demonstrated a strong consistence between the predicted survival probabilities and the actual survival rates for the respective time intervals in both cohorts. (H, K) DCA (Decision Curve Analysis) demonstrated the strong predictive performance of SRLS compared to other clinical predictors in both cohorts.

We assessed the occurrence of various clinical pathological factors in both the low and high-risk groups to examine the relationship between the risk model and clinical pathological characteristics in ICCA patients (Figure 6A). We found that more advanced TNM staging, more perineural invasion, more regional lymph node metastases, and more vascular invasion were more frequently associated with higher risk groups, which is consistent with a poorer prognosis in the high-risk group ((Figure 6B,6C and 6D).

Univariate and multivariate Cox regression analyses of risk scores in test and train cohorts were performed to exclude the interference of other clinical factors (Figure 6E,6F). In both univariate and multivariate cox regression analyses risk score was an independent significant risk factor for OS in both test and training cohorts, which demonstrated its strong independent predictive potential. It's worth noting that intrahepatic metastasis (HR = 2.417, 95% CI = 1.630-3.584, P < 0.001), vascular invasion (HR = 0.392, 95% CI = 0.263-3.584, P < 0.001), regional lymph node metastasis (HR = 4.098, 95% CI = 2.715-6.185, P < 0.001), and distal metastasis (HR = 3.754, 95% CI = 1.926-7.318, P < 0.001) were independent prognostic predictors for the OS of ICCA patients too. To assess the individual probabilities of 1-year, 2-year, and 3-year survival rates for each patient, a nomogram was developed by combining clinical pathological features and risk scores in both test and training cohorts (Figure S4A, S4B). Furthermore, the calibration curve in Figure 6G,6I demonstrated a strong consistence between the predicted survival probabilities and the actual survival rates for the respective time intervals. DCA (Decision Curve Analysis) demonstrated the strong predictive performance of SRLS compared to other clinical predictors (Figures 6H, 6J).

To further generalize the predictive efficacy of SRLS, when factors, such as sex, age, TNM stage, distal metastasis, bile tract stone disease, regional lymph node metastasis etc. are considered, the OS of the high-risk group was significantly worse than low-risk group too (all p < 0.05, Figure S4C). This suggests that SRLS can be widely used to predict the survival of patients with ICCA.

In addition, the mutational landscape of the high and low-risk groups was illustrated in oncoplots, which showed that missense variants were the predominant type of mutation in both subgroups. The high-risk group had the TTN gene as the most frequently mutated, whereas in the low-risk group, it was KRAS. These genes that were mutated in ICCA tissues may be crucial in explaining the differences in prognosis between different risk groups (Figure S5A, B).

### Prediction performance for immune landscape, immune therapy, stemness of SRLS in ICCA

**Figure 7:**
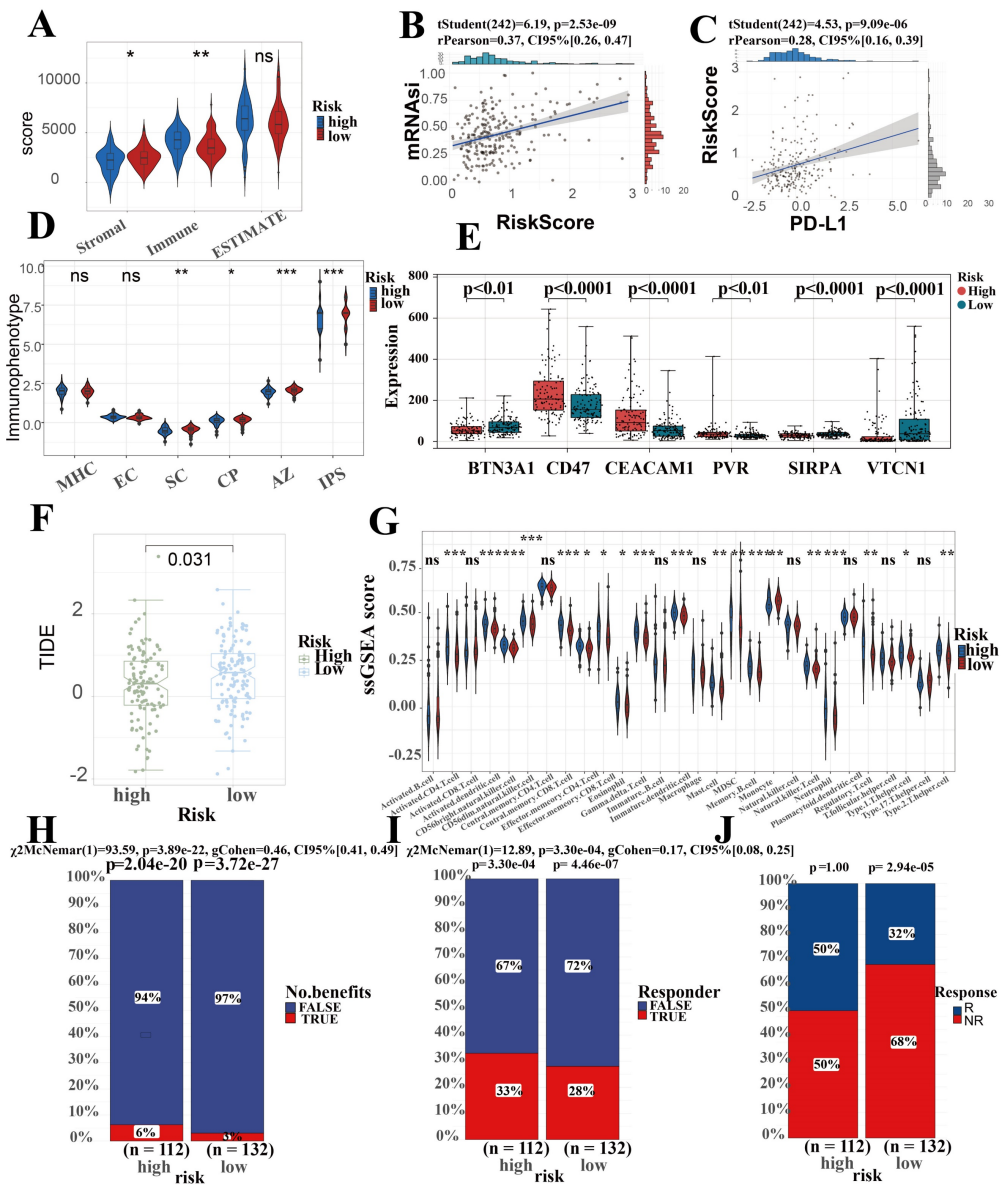
Prediction performance for immune landscape, immune therapy, stemness of SRLS in ICCA. (A) Estimate algorithm reveals higher proportion of immune cells and lower proportion of stromal cells in high-risk group. (B) A strong positive correlation between risk score and tumor stemness score. (C) A significant positive correlation between SRLS score and the expression of PD-L1. (D) The high-risk group was characterized by greater immune suppression and the low-risk group by greater immunogenicity based on immunophenotype score (IPS). (E) Significant differences observed in the expression of immune checkpoint genes among the two risk subgroups. (F) Lower TIDE prediction score suggests that high risk patients are more likely to benefit from immunotherapy. (G) Results of ssGSEA suggest an increased infiltration of almost all immune cells in the TME of the high-risk patients (H, I, J) Predictions of response to immunotherapy also showed higher rates in the high-risk group.

Micro-environmental alterations in tumors affect various behaviors and metabolism of tumors. Estimate algorithm reveals higher proportion of immune cells and lower proportion of stromal cells in high-risk group (Figure 7A). We found an obvious difference in the score of IPS which uses biomarkers of an immune response or immune tolerance to generates a z score that summarizes all the categories. High z scores in IPS indicating stronger immunogenicity. Thus, the high-risk group was characterized by greater immune suppression and the low-risk group by greater immunogenicity (Figure 7D). Furthermore, there were observable differences observed in the expression of immune checkpoint genes among the two subgroups. Specifically, BTN3A, CD47, CEACAM1, PVR, SIRPA, and VTCN1 were included in this analysis. Among these, CD47, CEACAM1, and PVR were higher in the high-risk group, while BTN3A, SIRPA, and VTCN1 were higher in the low-risk group (Figure 7E). Due to the great interest and application of PD-L1 in tumor therapy nowadays, we specifically explored (Figure 7C). This suggests that the differences in immune checkpoint expression could potentially serve as therapeutic targets for ICCA patients at different risk levels.

In Fu-ICCA cohort, we found a strong positive correlation between risk score and tumor stemness score, which implicates that SRLS exhibits good performance in predicting the degree of tumor differentiation and tumor stemness (Figure 7B).

In order to assess the immune infiltration more broadly and specifically, we took ssGSEA to assess the infiltration of immune cells, which suggest an increased infiltration of almost all immune cells in the TME of the high-risk patients (Figure 7G). Further EPIC algorithm revealed that Correlation between risk score and level of immune cell infiltration (Figure S3A). Specifically, we discovered that the risk scores were negatively correlated with the expression of T cells CD8 (R = -0.26, p = 0.023), B cells naïve (R = -0.47, p = 1.5e-05), dendritic cells resting (R = -0.38, p = 6e-04), monocytes (R = -0.23, p = 0.046), plasma cells (R = -0.27, p = 0.017), and T cell CD4 memory resting (R = -0.45, p = 5.5e-05), while they were positively correlated with macrophages M0 (R = 0.5, p = 3.3e-06), macrophages M2 (R = 0.41, p = 0.00024), and neutrophils (R = 0.25, p = 0.033). Overall, these results confirm the impact of SRLS on ICCA patients.

TIDE scores can provide insight into whether a patient will respond to immune checkpoint blockades, including anti-PD1 and anti-CTLA4. Higher TIDE prediction scores represent a higher likelihood of immune evasion, suggesting that patients are less likely to benefit from immunotherapy. Our results showed that TIDE scores were significantly lower in the high-risk group than in the low-risk group, suggesting that high-risk group more likely to benefit from immunotherapy (Figure 7F). Predictions of response to immunotherapy also showed higher rates in the high-risk group (Figure 7H, 7I and 7J).

### Identification of hub lncRNA-miRNA-mRNA axis involving in senescence in ICCA

**Figure 8:**
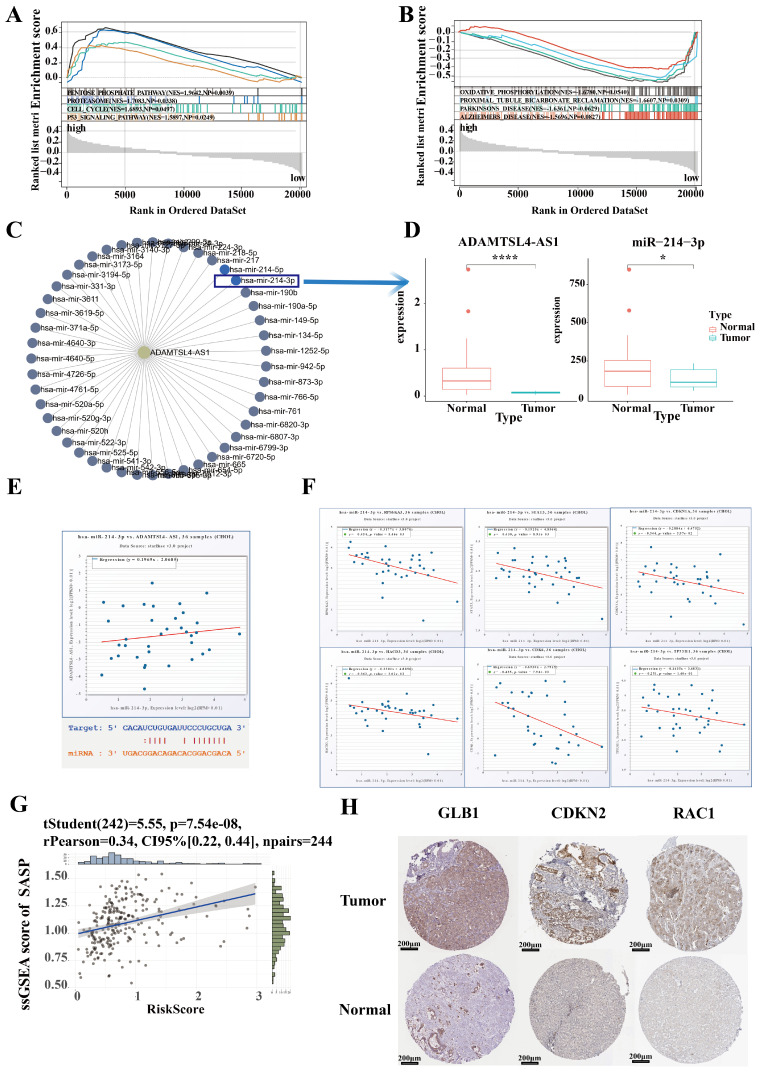
Identification of hub lncRNA-miRNA-mRNA axis involving in senescence in ICCA. (A, B) Characterization of the difference of biological function between high and low risk groups via GSEA. (C) The lncRNA-miRNA regulatory network was constructed to discover the target miRNA of ADAMTSL4-AS1. (D)ADAMTSL4-AS1 and miR-214-3P were significantly decreased in tumor samples in the TCGA-CHOL cohort. (E) A positive correlation between the expression levels of ADAMTSL4-AS1 and miR-214-3p; The predicted binding sites for both are shown. (F) A series of target mRNAs closely related to senescence, such as CDKN1A(p21), HACD3, CDK6. (G) Risk score were significantly and positively correlated with SASP score. (H) The expression of senescence-related proteins that is targeted by miR-214 was significantly different in tumor tissues and normal tissues.

To further characterize differences in biological function between high and low risk groups, we identified 2,000 differential genes between high and low risk groups and used them to characterize the difference of biological function between high and low risk groups via GSEA (Figure 8A, 8B). ICCA samples of High risk were significantly enriched in pentose phosphate pathway, proteasome pathway, cell cycle pathway, p53 signaling pathway. ICCA samples of Low risk were significantly enriched in oxidative phosphorylation pathway, proximal tubule bicarbonate reclamation pathway, parkinsons disease pathway, Alzheimers disease pathway.

To specifically validate the mechanism of SRLS in ICCA, we plan to establish representative molecular biological mechanisms to personalize the role of SRLS. The lncRNA-miRNA regulatory network was first constructed to discover the target miRNA of ADAMTSL4-AS1 (Figure 8C). Notably, a previous study has demonstrated that miR-214-3p involved in cellular senescence 28. Interestingly, we found that ADAMTSL4-AS1D and miR-214-3P were significantly decreased in tumor samples in the TCGA-CHOL cohort (Figure 8D). The predicted binding sites for both are shown in Figure 8E. Further linear regression showed a positive correlation between the expression levels of ADAMTSL4-AS1 and miR-214-3p (Figure 8E). Next, we predicted a series of target mRNAs for the miRNAs and selected targets that are closely related to senescence, such as CDKN1A (p21), HACD3, CDK6 (Figure 8F). Several potential lncRNA-miRNA-mRNA axes involved in ICCA by regulating senescence were identified.

We performed ssGSEA calculating SASP scores for each sample and found that risk score was significantly and positively correlated with SASP score, suggesting that SRLS may exhibit senescence characteristics and behaviors by promoting senescence associated secretory phenotypes and further participate in tumorigenesis of ICCA (Figure 8G). To verify the changes in the expression levels of senescence-associated mRNA targets in ICCA, we obtained the corresponding immunohistochemistry specimens from the HPA (Human protein atlas) database and found that the expression of these proteins was significantly different in tumor tissues and normal tissue (Figure 8H).

### Predicting chemotherapeutic drug sensitivity in ICCA using SRLS

**Figure 9:**
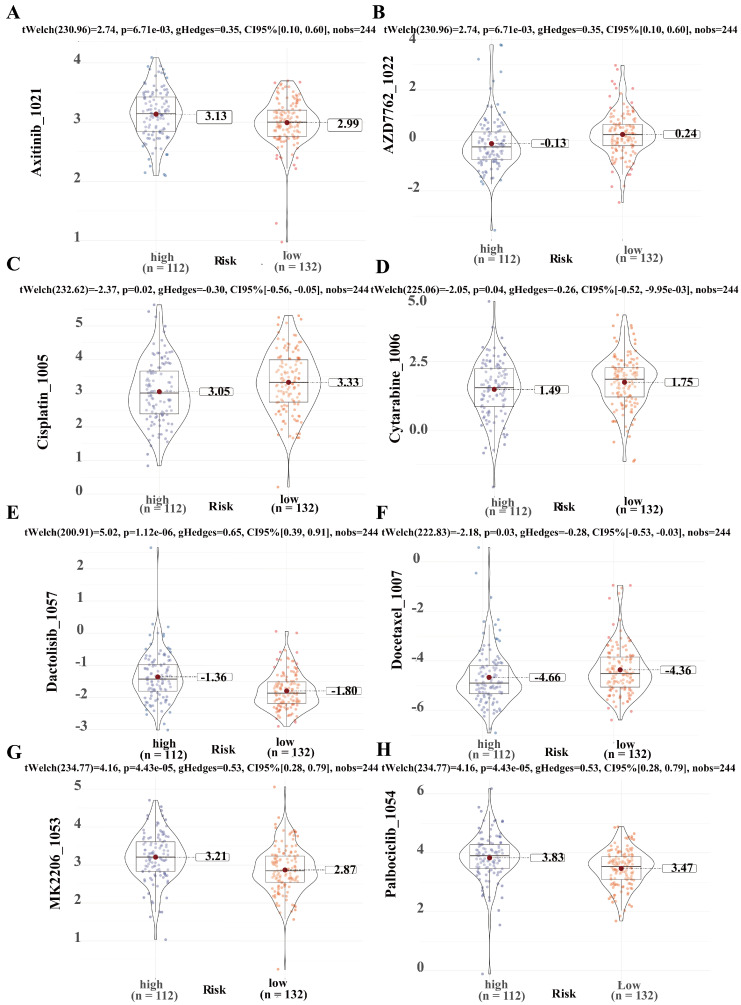
Predicting chemotherapeutic drug sensitivity in ICCA using SRLS. (A, B, C, D, E, F, G, H) Common chemotherapeutic drug sensitivity in ICCA between high and low risk groups including Axitinib, AZD7762, Cisplatin, Cytarabine, Dactolisib, Docetaxel, MK2206 and Palbciclib.

To predict the application value of SRLS in chemotherapy for ICCA patients, we evaluated the relationship between the sensitivity and risk score of commonly used ICCA chemotherapeutic agents. We discovered that the resistance to AZD7762 (Figure 9B), Cisplatin (Figure 9C), Cytarabine (Figure 9D), Docetaxel (Figure 9F) in the high-risk group was higher than that in the low-risk group, while patients in the high-risk group were less resistant to Axitinib (Figure 9A), Dactolisib (Figure 9E), MK2206 (Figure 9G), Palbciclib (Figure 9H) than those in the low-risk group. These results suggest that SRLS characteristics are effective in predicting the sensitivity of ICCA patients to common chemotherapeutic agents, thus providing guidance for chemotherapy dosing in both high- and low-risk patients.

## Discussion

Hepatocellular carcinoma has been the most common primary liver malignancy, followed by ICCA as the second most prevalent, and the morbidity and mortality rates of ICCA are consistently increasing worldwide 1. Researches into the mechanisms of ICCA occurrence have revealed that the death of intrahepatic bile duct cells because of senescence leads to the development of tumors in a tumor microenvironment dominated by senescence 31. Recent research has additionally shown that cellular senescence has a significant impact on the regulation of different facets of cancer, such as the development of cancer, types of cancer, the immune response, the metastasis, and treatments 32. Nevertheless, the perception of the leading molecular mechanisms for the generation and progression of ICCA, because of cellular senescence, remains incomplete.

In this study, the focus was on constructing and validating SRLS models of ICCA based on senescence-associated genes and lncRNAs. We identified SRG and SR-lncRNAs based on the Fu-ICCA cohort. Afterwards, an unsupervised clustering machine learning approach was utilized to identify and classify senescence-associated lncRNAs and their molecular subtypes into 2 distinct categories. The two clusters showed some heterogeneity in terms of immune cell infiltration, immune checkpoints and tumor stemness, which opens the possibility of personalizing the diagnosis and treatment of ICCA patients. In terms of clinical characteristics and prognosis between the two clusters, cluster 1 exhibits poorer prognosis, more advanced TNM staging as well as more perineural invasion. More importantly, we found in GSVA that cluster 1 showed more features of senescence. It is necessary to highlight that the 20 SR-lncRNAs we selected are described based on their expression levels linked to SRGs, and further investigation is in need to determine if these SR-lncRNAs have expression regulatory mechanisms with SRGs.

To more accurately and specifically describe and characterize the performance of senescence in ICCA, we fitted 113 predictive models based on machine learning using the LOOCV framework. Based on the performance in the four cohorts we selected the best model with the highest average AUC value. Next, a risk score formula based on the expression of key lncRNAs were developed which includes TMEM75, Cr10orf55, AL627171.2, AL136115.1, PAXIP1-AS2, LOH12CR2, DNAH10OS, ADAMTSL4-AS1, AC079210.1, AC040977.1, and AC112721.1, the senescence-associated lncRNA signatures (SRLS). Among those lncRNAs, some findings have indicated that LOH12CR2, a cancer immune-related lncRNA, linked to antibiotics, cytokines, interleukins, antigen processing and presentation, natural killer cytotoxicity, TCR signaling, chemokines, and interleukin receptor pathways 33. Also, Umakanta Swain et al. discovered that PAXIP1-AS2 is a new regulator of translesion DNA synthesis (TLS), implying that the absence of PAXIP1-AS2 might contribute to oncogenesis through inhibiting the repair of DNA damage 34. This suggests the importance of these lncRNAs in tumor.

Next, we validated the predictive power of SRLS. On the one hand we validated the reliability of the risk stratification and prognostic models in internal and external cohorts. On the other hand, we validated SRLS as an independent risk factor for OS in ICCA patients by univariate and multivariate cox regression. The nomogram and DCA reconfirmed the robust predictive and stratification capabilities of SRLS. And after generalization, this ability holds across most subgroups.

Cellular senescence has traditionally been recognized as an anti-tumor mechanism in response to oncogenic stress. Recent studies have shown that senescent cells can also create an immunosuppressive, vasodilatory, and pro-inflammatory tumor microenvironment through SASP, thereby promoting tumor growth and progression. Senescence-associated secretory phenotype (SASP) refers to the large number of factors secreted by senescent cells, including pro-inflammatory cytokines and chemokines, growth regulators, angiogenic factors, and matrix metalloproteinases (MMPs) 35. Interestingly, we noted a significant positive correlation between risk scores and ssGSEA scores for SASP, and thus we speculate that high levels of SASP are responsible for the poor prognosis of the high-risk group.

Heterogeneity in the immune micro environment between high- and low-risk groups may arise due to senescence, and subsequently affect tumor behavior and metabolism. We predicted immune landscape, immunotherapy, chemotherapy, and tumor stemness based on SRLS. IPS is the indicative of immunogenicity, which including four essential gene categories that determine immunogenicity: effector cells, immunosuppressive cells, MHC molecules, and immunomodulators 36, 37. Accordingly, we found that the high-risk group showed more immunosuppression, while the low-risk group was characterized by stronger immunogenicity. In contrast, ssGSEA revealed that most of the immune cells infiltrated in the high-risk group more. The contradiction might be attributed to the strong positive correlation between M0, M2 macrophages and risk scores. M2 macrophages are similar in phenotype to TAMs (tumor associated macrophages), which promote tumor growth and metastasis, and are associated with poor prognosis of tumors 38. There is also evidence that the accumulation of M0 macrophages in tumors is associated with a poorer prognosis 39. Recent ICCA immune cell profiles also indicated that TAMs (tumor associated macrophages) play a more prominent role in ICCA TME (Tumor micro-environment) compared to other myeloid cells and should be deemed as a primary target for immunotherapy in ICCA 40. Immune checkpoint inhibitors (ICIs) are the fourth most common cancer treatment method, following surgery, chemotherapy, and radiotherapy, and help combat immune evasion. Lower TIDE score in high-risk group suggests that the high-risk group is more likely to benefit from immunotherapy. Furthermore, the differences in immune checkpoint and chemotherapeutic drug sensitivity could potentially serve as therapeutic targets for ICCA patients at different risk levels.

At the genomic level, TP53 mutations appeared more frequently in the high-risk group, whereas the frequency of KRAS mutations was most frequent in the low-risk group. TP53 is involved in cellular senescence through various signaling pathways. For instance, the p53/p21 pathway is involved in cell proliferation arrest and tumor suppression 41. Thus, mutations of the TP53 gene in the high risk may lead to loss of tumor suppressor function and contribute to ICCA progression.

Research has shown that long non-coding RNAs (lncRNAs) act as epigenetic factors in cancer initiation and development 42. Studies have also centered around their connection to targets which is most often a microRNA. Li found SNHG1, a long non-coding RNA, acting as a ceRNA (competing endogenous RNA) for miR-140, promotes the expression of TLR4 and activates the NF-ĸB signaling pathway, thus regulating the growth and tumorigenesis of cholangiocarcinoma. We first established the lncRNA-microRNA network. Among them, miR-214-3p was previously reported to promote endothelial cell senescence and inhibit cell proliferation 28. We then identified mRNA targets downstream of microRNAs that are associated with aging. The activation of cyclin D-CDK4/6 is a crucial factor in developing various cancers. To combat this, small-molecule inhibitors of CDK4/6 have been used successfully to treat hormone receptor-positive breast cancers and are currently being tested in clinical trials for other types of tumors 43. CDK6 showed a significant negative correlation with miR-214-3p, suggesting that miR-214-3p inhibits tumorigenesis by degrading CDK6. When miR-214-3p levels decreased, high levels of CDK6 promoted tumor initiation and progression, which is essentially a reverse aging process. However, the pleiotropic and bidirectional nature of cellular senescence often cannot be ignored in tumors. Another opposite representative downstream target is CDKN1A (p21), who has been proved to significantly accumulates during cellular senescence 44. CDKN1A is found to negatively correlated with miR-214-3p. When miR-214-3p levels decreased, CDKN1A accumulated, which led to the onset of cellular senescence and more SASP. Cellular senescence is differentially affected in the two examples above, yet both lead to tumor initiation and progression.

There are also limitations in our study, as the general conclusions were based on analyzing RNA-seq data within the database and were not confirmed in an extensive clinical group. Additionally, the primary expression of SR-lncRNA in these cells in ICCA is not clear, and experimental studies are necessary to understand the precise mechanisms of how SR-lncRNAs influence the immune response and patient prognosis in ICCA. Moving forward, our commitment would be remaining steadfast in closely monitoring these issues and diligently striving to address them.

## Conclusion

To summarize, we developed a novel senescence-based prognostic signature. SRLS can favorably predict the prognosis, risk stratification, immune landscape and immunotherapy response of patients with ICCA and consequently guide individualized treatment. In addition, these findings hold considerable implications for comprehending the clinical features and future outcomes of ICCA, and further investigation is necessary to unravel the biological mechanisms underlying their specific functions.

## Supplementary Material

Supplementary figures and tables.

## Abbreviations

ICCA: intrahepatic cholangiocarcinoma
SR-lncRNAs: senescence-associated long non-coding RNAs
miRNA: microRNA
SRGs: senescence-associated genes
ssGSEA: single sample gene set enrichment analysis
IPS: immunophenotype score
TIDE: tumor immune dysfunction and exclusion
SASP: senescence-associated secretory phenotype
NODE: National Omics Data Encyclopedia
CDF: Cumulative distribution function
GSVA: Gene Set Variation Analysis
SRLS: senescence-related lncRNA signature
LOOCV: Leave One Out Cross-Validation
LASSO: least absolute shrinkage and selection operator
HBsAg: hepatitis B surface antigen
OS: overall survival
HCC: hepatocellular carcinoma
MSI: Microsatellite Instability
mRNAsi: mRNA Stemness index
IC50: the half-maximal inhibitory concentration
AUC: area under curve
HR: hazard ratio
CI: Confidence interval
DCA: Decision Curve Analysis
HPA: Human protein atlas
TAMs: Tumor associated macrophages
TME: Tumor micro-environment
ICI: Immune checkpoint inhibitor
ceRNA: competing endogenous RNA
