# Supplementary material for: Machine Learning-based Development and Validation of a Cell Senescence Predictive and Prognostic Signature in Intrahepatic Cholangiocarcinoma
Source: J Cancer. 2024 Mar 25;15(9):2810–28. doi: 10.7150/jca.92698 (PMC10988306; doi:10.7150/jca.92698)
Supplement: Supplementary file 1 — Supplementary figures and tables. [file jcav15p2810s1.pdf]

Figure S1 Differential expression of 19 SR-lncRNAs between clusters

Figure S2 (A)Relationship between clinical characteristics and SRLS scores. (B)Co-expression relationships between 20 SR-lncRNAS.

Figure S3 (A) EPIC algorithm reveals correlation between immune cell infiltration and SRLS scores. (B)Volcano plot showing differential genes between high and low risk groups based on limma package. (C)Venn diagram showing intersection of SRGs with differential genes. (D)Clustering heatmap showing specific up-regulated differential genes vs. down-regulated differential genes.

Figure S4 (A,B)Nomograms developed by combining clinicopathological features and risk score in both test and training cohorts to assess the individual probabilities of 1-year, 2-year, and 3-year survival rates. (C)Subgroup survival analysis to assess the clinical applicability of SRLS.

Figure S5 (A,B)Mutational landscapes of different SRLS subgroups.

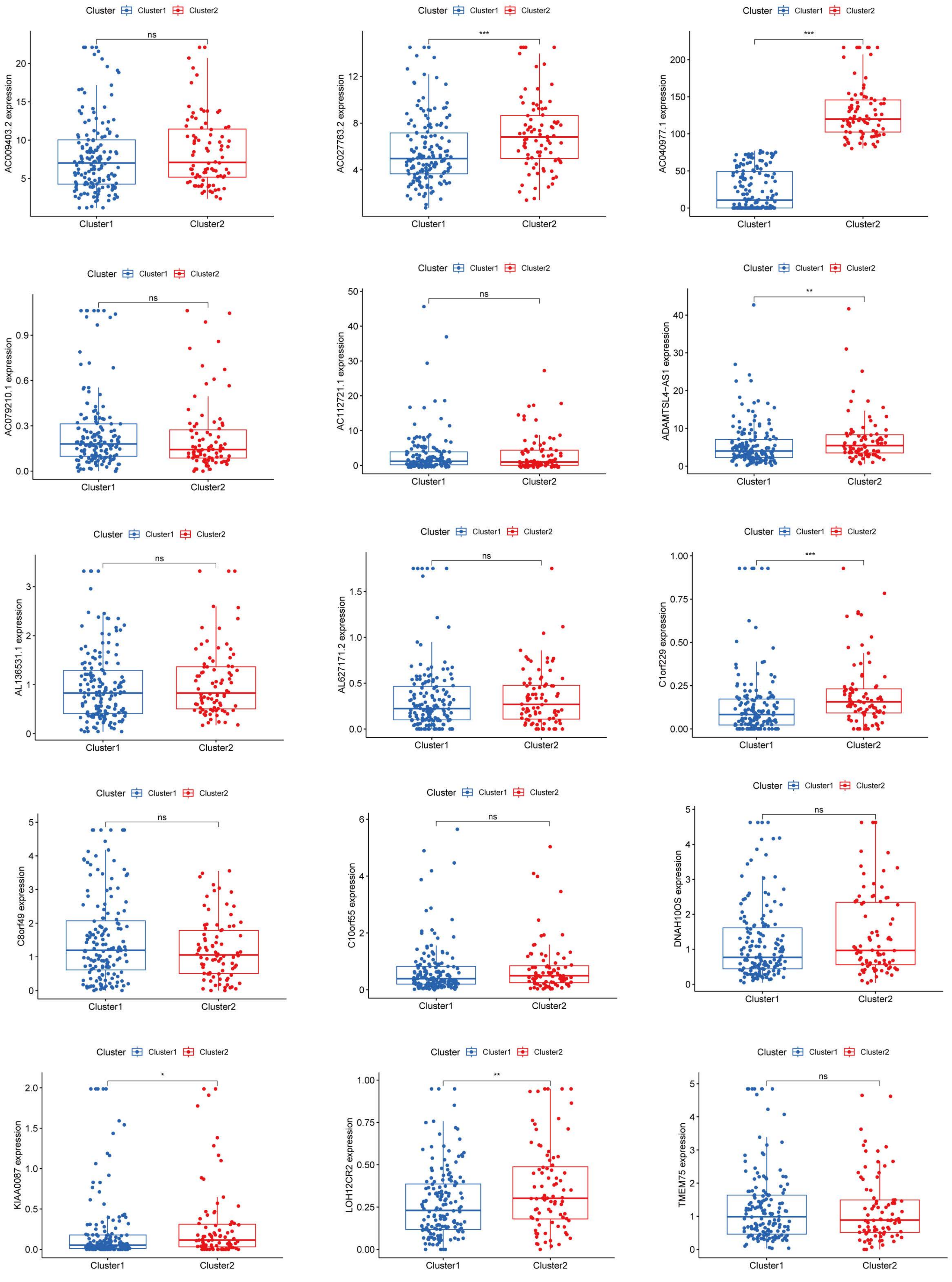

Cluster Cluster1 Cluster2
AC009403.2 expression
ns
AC027763.2 expression
***
AC040977.1 expression
***
AC079210.1 expression
ns
AC112721.1 expression
ns
ADAMTSL4-AS1 expression
**
AL136531.1 expression
ns
AL627171.2 expression
ns
C1orf229 expression
***
C8orf49 expression
ns
C10orf55 expression
ns
DNAH10OS expression
ns
KIAA0087 expression
*
LOH12CR2 expression
**
TMEM75 expression
ns
Cluster1
Cluster2

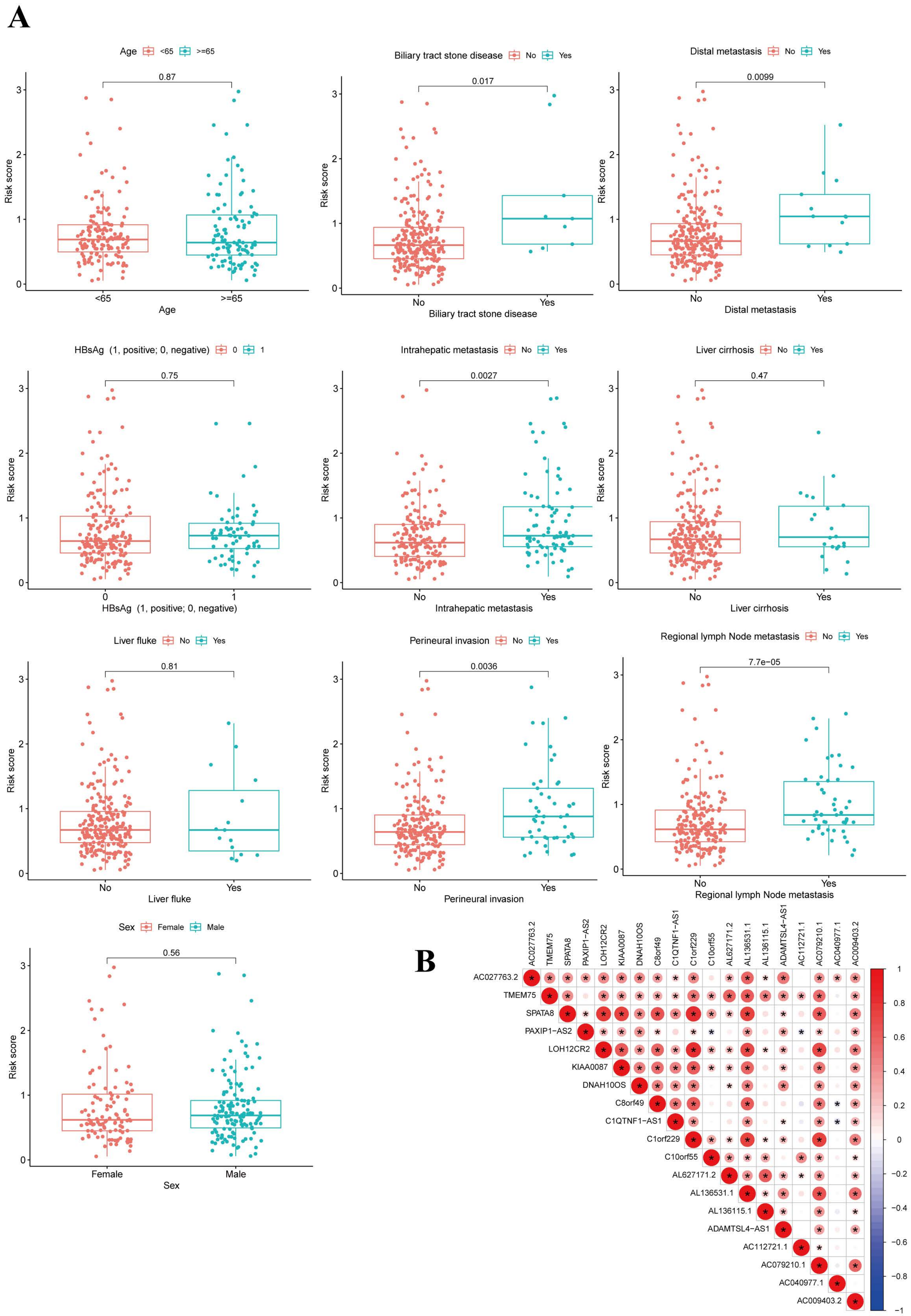

A
Age <65 >=65
0.87
Risk score
Age
Biliary tract stone disease No Yes
0.017
Biliary tract stone disease
Distal metastasis No Yes
0.0099
Distal metastasis
HBsAg (1, positive; 0, negative) 0 1
0.75
HBsAg (1, positive; 0, negative)
Intrahepatic metastasis No Yes
0.0027
Intrahepatic metastasis
Liver cirrhosis No Yes
0.47
Liver cirrhosis
Liver fluke No Yes
0.81
Liver fluke
Perineural invasion No Yes
0.0036
Perineural invasion
Regional lymph Node metastasis No Yes
7.7e−05
Regional lymph Node metastasis
Sex Female Male
0.56
Sex
B
AC027763.2
TMEM75
SPATA8
PAXIP1−AS2
LOH12CR2
KIAA0087
DNAH10OS
C8orf49
C1QTNF1−AS1
C1orf229
C10orf55
AL627171.2
AL136531.1
AL136115.1
ADAMTSL4−AS1
AC112721.1
AC079210.1
AC040977.1
AC009403.2

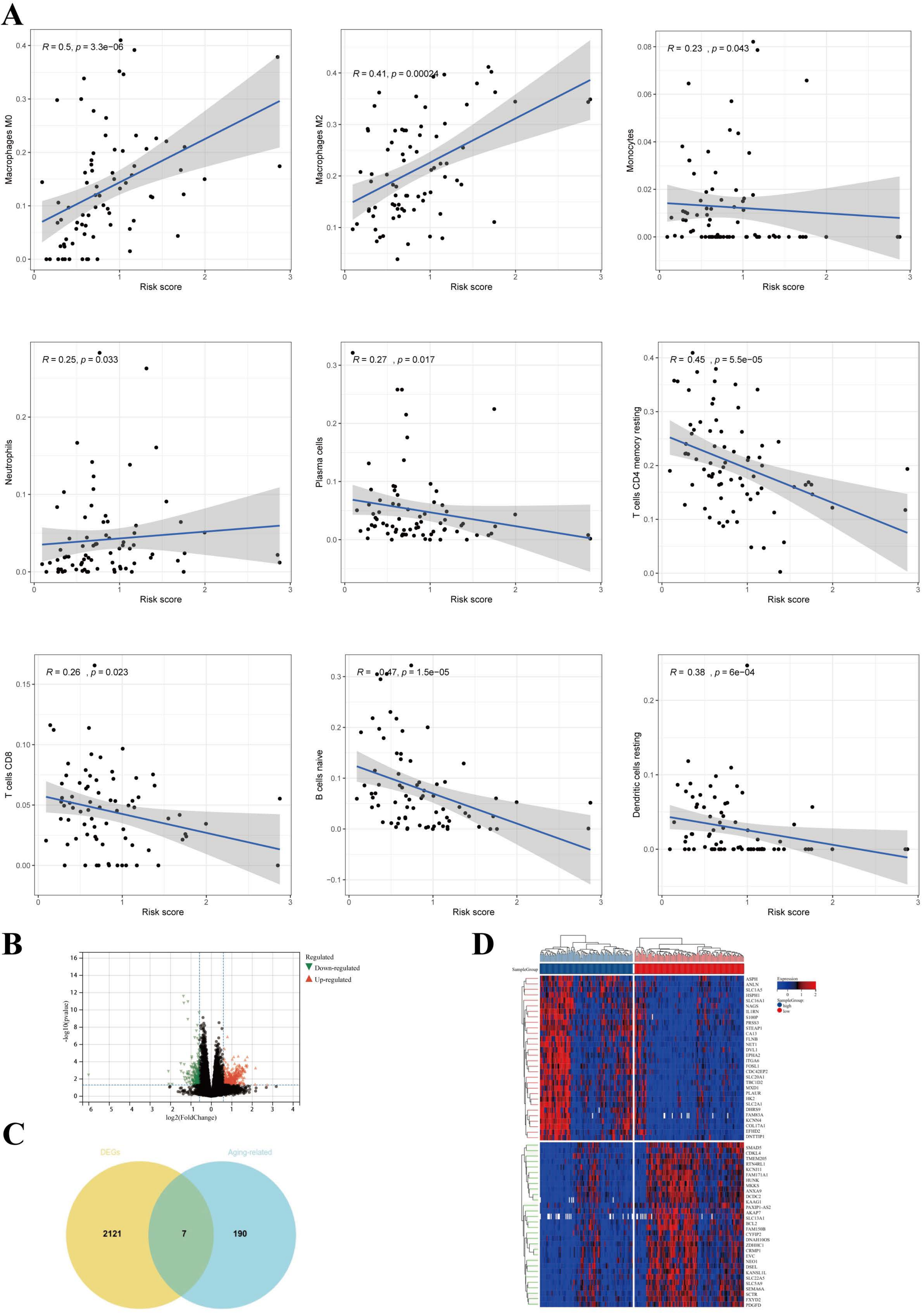

A
R = 0.5, p = 3.3e−06
Macrophages M0
Risk score
R = 0.41, p = 0.00024
Macrophages M2
Risk score
R = 0.23 , p = 0.043
Monocytes
Risk score
R = 0.25, p = 0.033
Neutrophils
Risk score
R = 0.27 , p = 0.017
Plasma cells
Risk score
R = 0.45 , p = 5.5e−05
T cells CD4 memory resting
Risk score
R = 0.26 , p = 0.023
T cells CD8
Risk score
R = 0.47, p = 1.5e−05
B cells naive
Risk score
R = 0.38 , p = 6e−04
Dendritic cells resting
Risk score
B
Regulated
Down-regulated
Up-regulated
-log10(pvalue)
log2(FoldChange)
C
DEGs
Aging-related
2121
7
190
D
SampleGroup
Expression
SampleGroup:
high
low
ASPH
ANLN
SLC1A5
HSPH1
SLC16A1
NAGS
IL1RN
S100P
PRSS3
STEAP1
CA13
FLNB
NET1
DVL1
EPHA2
ITGA6
FOSL1
CDC42EP2
SLC20A1
TBC1D2
MXD1
PLAUR
HK2
SLC2A1
DHRS9
FAM83A
KCNN4
COL17A1
EFHD2
DNTTIP1
SMAD5
CDKL4
TMEM205
RTN4RL1
KCNJ11
FAM171A1
HUNK
MKKS
ANXA9
DCDC2
KAAG1
PAXIP1-AS2
AKAP7
SLC13A1
BCL2
FAM150B
CYFIP2
DNAH10OS
ZDHHC1
CRMP1
EVC
NEO1
DSEL
KANSL1L
SLC22A5
SLC5A9
SEMA6A
SCTR
FXYD2
PDGFD

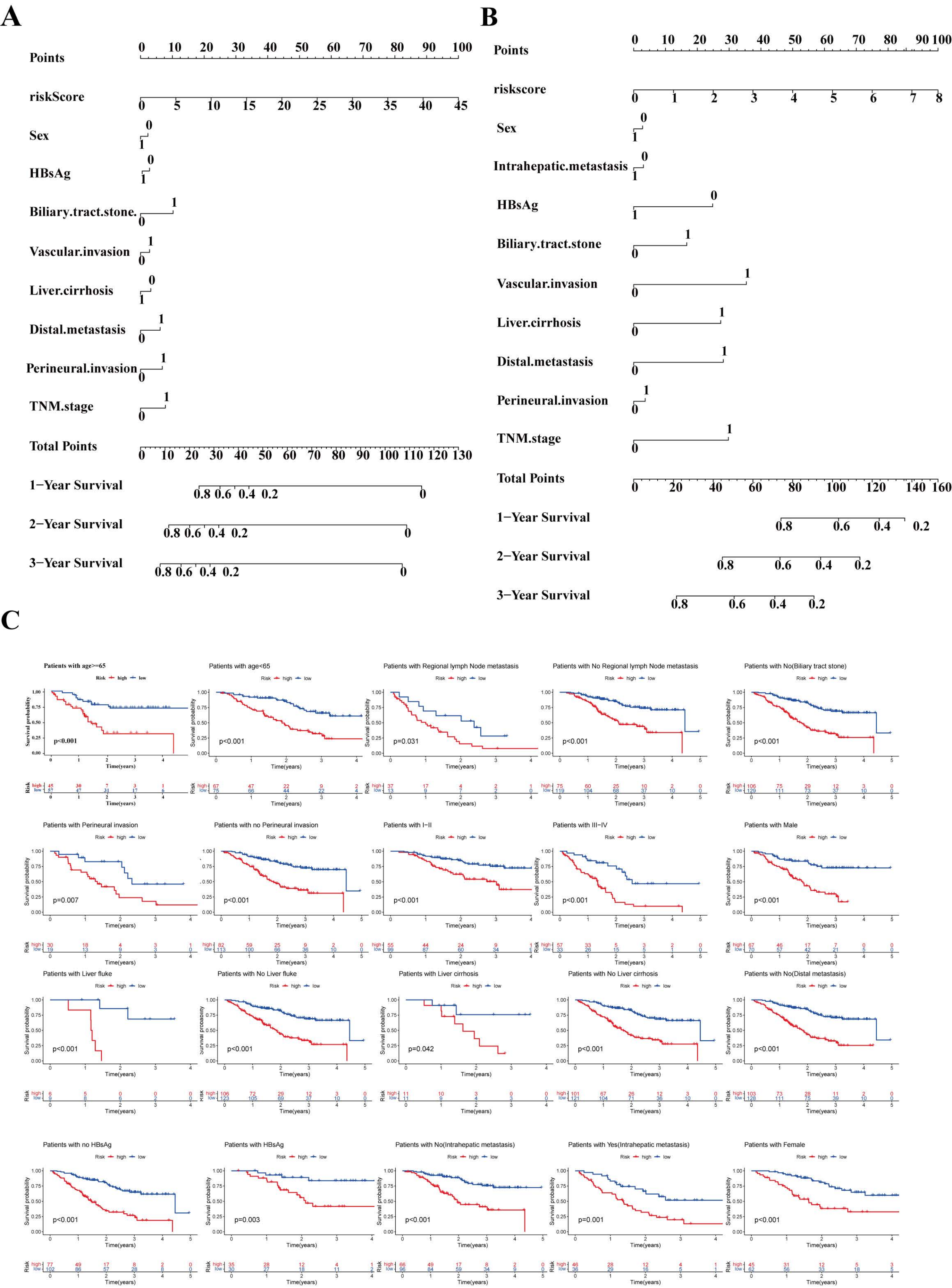
A
Points
riskScore
Sex
HBsAg
Biliary.tract.stone.
Vascular.invasion
Liver.cirrhosis
Distal.metastasis
Perineural.invasion
TNM.stage
Total Points
1−Year Survival
2−Year Survival
3−Year Survival
B
Points
riskscore
Sex
Intrahepatic.metastasis
HBsAg
Biliary.tract.stone
Vascular.invasion
Liver.cirrhosis
Distal.metastasis
Perineural.invasion
TNM.stage
Total Points
1−Year Survival
2−Year Survival
3−Year Survival
C
Patients with age>=65
p<0.001
Patients with age<65
p<0.001
Patients with Regional lymph Node metastasis
p=0.031
Patients with No Regional lymph Node metastasis
p<0.001
Patients with No(Biliary tract stone)
p<0.001
Patients with Perineural invasion
p=0.007
Patients with no Perineural invasion
p<0.001
Patients with I−II
p<0.001
Patients with III−IV
p<0.001
Patients with Male
p<0.001
Patients with Liver fluke
p<0.001
Patients with No Liver fluke
p<0.001
Patients with Liver cirrhosis
p=0.042
Patients with No Liver cirrhosis
p<0.001
Patients with No(Distal metastasis)
p<0.001
Patients with no HBsAg
p<0.001
Patients with HBsAg
p=0.003
Patients with No(Intrahepatic metastasis)
p<0.001
Patients with Yes(Intrahepatic metastasis)
p=0.001
Patients with Female
p<0.001
Risk high low
Survival probability
Time(years)

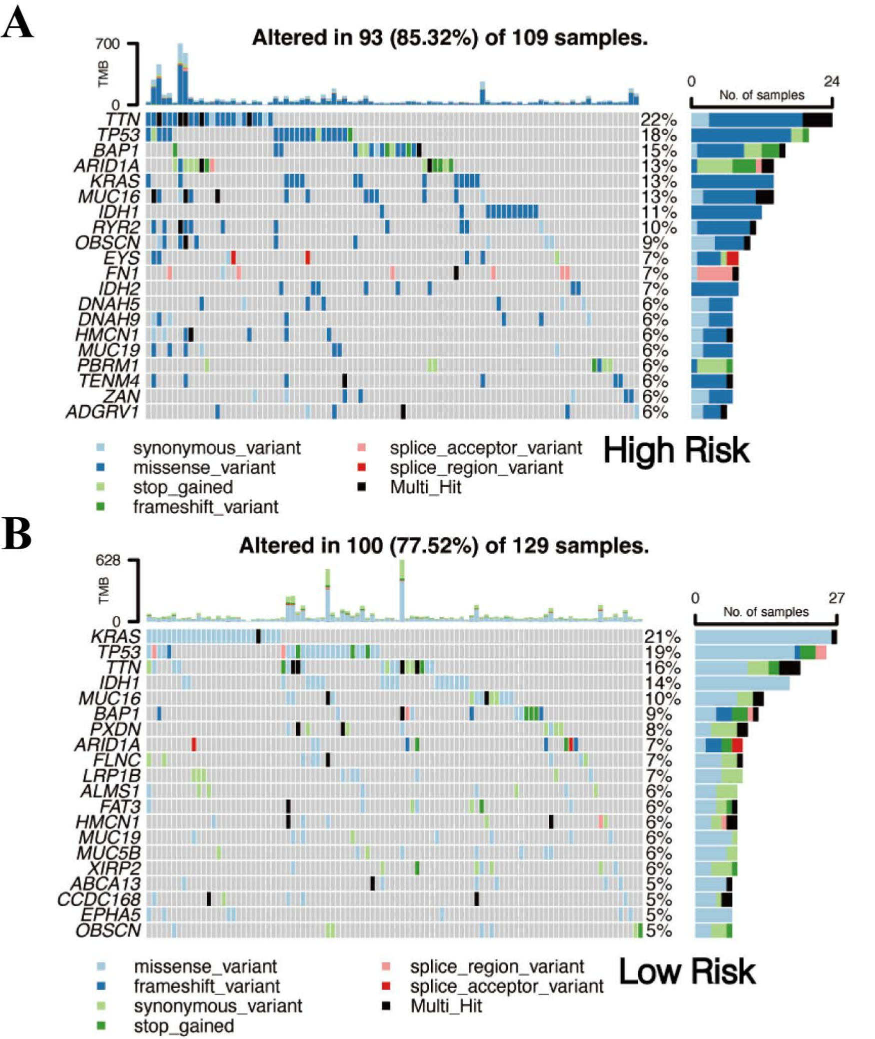
A
Altered in 93 (85.32%) of 109 samples.
700
TMB
0
No. of samples
24
TTN 22%
TP53 18%
BAP1 15%
ARID1A 13%
KRAS 13%
MUC16 13%
IDH1 11%
RYR2 10%
OBSCN 9%
EYS 7%
FN1 7%
IDH2 7%
DNAH5 6%
DNAH9 6%
HMCN1 6%
MUC19 6%
PBRM1 6%
TENM4 6%
ZAN 6%
ADGRV1 6%
synonymous_variant
missense_variant
stop_gained
frameshift_variant
splice_acceptor_variant
splice_region_variant
Multi_Hit
High Risk
B
Altered in 100 (77.52%) of 129 samples.
628
TMB
0
No. of samples
27
KRAS 21%
TP53 19%
TTN 16%
IDH1 14%
MUC16 10%
BAP1 9%
PXDN 8%
ARID1A 7%
FLNC 7%
LRP1B 7%
ALMS1 6%
FAT3 6%
HMCN1 6%
MUC19 6%
MUC5B 6%
XIRP2 6%
ABCA13 5%
CCDC168 5%
EPHA5 5%
OBSCN 5%
missense_variant
frameshift_variant
synonymous_variant
stop_gained
splice_region_variant
splice_acceptor_variant
Multi_Hit
Low Risk
